# Qualitative investigation of barriers to providing an electronic hospital to community pharmacy referral service for discharged patients

**DOI:** 10.1371/journal.pone.0283836

**Published:** 2023-03-31

**Authors:** Sarah M. Khayyat, Hamde Nazar

**Affiliations:** 1 Department of Clinical Pharmacy, Faculty of Pharmacy, Umm Al-Qura University, Makkah, Saudi Arabia; 2 School of Pharmacy, Newcastle University, Newcastle upon Tyne, United Kingdom; University of the Witwatersrand Johannesburg Faculty of Health Sciences/ Nnamdi Azikiwe University Awka, SOUTH AFRICA

## Abstract

There has been a sustained interest in transfer of care (ToC) services, from hospital to home, in the past twenty years. In England, an electronic referral (e-referral) service from hospital to community pharmacy has been provided since 2014. However, to date, there has been little information about service implementation and delivery. This study investigates the barriers to this referral process in hospital and community pharmacy settings, and barriers to providing subsequent community pharmacy interventions from the perspectives of the service leaders (SLs), hospital pharmacy staff (HPS) and community pharmacists (CPs). Semi-structured face-to-face or telephone interviews were conducted with the key informants from two tertiary hospitals and nine pharmacies. The Consolidated Framework for Implementation Research (CFIR) tool informed the data collection tools and data analysis. A total of three SLs, ten HPS and nine CPs were interviewed. Data analysis identified various barriers to the provision of the e-referral service. Some were related to (1) patient engagement, e.g., patients’ awareness/acceptance of the service, (2) the SLs and other National Health Service hospitals, e.g., lack of monitoring of the service progress, (3) the HPS, e.g., resource limitations, or (4) the CPs, e.g., lack of understanding/appreciation of the service. In-depth understanding of barriers related to the provision of e-referral service are essential to drive improvement and facilitate wider diffusion and adoption. The use of implementation science and behaviour change model as a lens to assess this service enables the identification of certain behaviours that can be modified to produce the required change to drive better implementation and delivery.

## 1. Introduction

Worldwide, there has been a sustained interest in transfer of care (ToC) services, from hospital to home, in the past twenty years [1]. In the United States (US), many services and disease management programmes have targeted patients with chronic diseases to provide patient support, education, counselling, monitoring, and medication oversight through the continuum of care following hospital discharge [2]. The Diabetes Self-Management Education (DSME) programme [3], the Medication-Therapy Management (MTM) service [4], and the Automated Telephone Self-Management (ATSM) support [5] are some examples of the US post-discharge interventions. Similarly, in the United Kingdom (UK), different ToC services have been delivered to discharged patients in which hospital pharmacists refer them to community pharmacists for follow-up care. These ToC services aim to provide out-of-hospital support and improve quality and quantity of communication between healthcare providers [1, 6]. Examples of these services include the Refer to Pharmacy (R2P) service, the Newcastle-Upon-Tyne Hospitals Trust electronic referral (NUTH e-referral) service, and the Connect with Pharmacy (CwP) service [6]. Moreover, in February 2021, the Discharge Medicines Service (DMS) became a new essential ToC service provided by all community pharmacy contractors in England. In the DMS, when patients are discharged, hospitals will digitally refer them to their community pharmacy for additional support and follow-up care about their medicines [7]. It is believed that the service will benefit patients taking high-risk medicines, those who have had changes made to their medicines regimen during the hospital stay, and those who have been prescribed new medicines [8].

This study focuses on one of the key functions of the process evaluation of the British Medical Research Council (MRC) framework [9]; which is the context affecting the implementation process and outcomes of the ToC or e-referral services [10]. Damschroder *et al*. defined the ‘context’ as a set of circumstances or unique factors that surround a particular implementation effort [11]. These circumstances or factors may act as barriers or facilitators to the service implementation or its effect [10]. Studies have found that evaluation of the implementation process considering the context helps in assessing the effectiveness of the implemented intervention and understanding reasons for potential success or failure of a service, with more focus on weak links in the causal chain and process that could be strengthened to meet patients’ needs [9–11]. Therefore, it is believed that the implementation of any service or intervention should consider three key elements; these are (1) having theories and empirical research that focus on exploring and understanding different healthcare systems, behaviours, and practices to influence successful implementation, (2) identifying and developing strategies to address barriers or facilitators to the service implementation in a specific context, and (3) understanding the process of implementation itself of how the service, in this case: the e-referral service, was implemented and caused change, e.g., evaluating its delivery process [12].

Specific contextual factors (barriers) have been previously reported in pharmacy interventions. This included poorly developed pharmacist-doctor relationships, poor public perception/experience of the provided services, pharmacists’ education/skills/motivation to provide the service, poor communication between healthcare professionals during the ToC, and the lack of time to implement a service [13, 14]. To address these barriers and improve the design of the intervention and its evaluation, researchers are recommended to apply theoretical frameworks or models to identify certain behaviours/beliefs that can be changed [15]. Examples of some desired behaviours include motivating healthcare professionals to use new technology, improving workflow in community pharmacies, and engaging healthcare professionals in promoting and offering new technologies/services to hospitalised patients [16–18].

Although a range of previous ToC initiatives have been piloted or implemented in the UK, there is still little information collected about these services from an implementation and delivery perspective that would contextualise any service activity or outcome data [19]. For instance, a recent narrative review identified different potential elements of an effective intervention to improve the ToC and reduce healthcare utilisation in patients with diabetes. However, authors emphasised that the generalisability of their findings and the replication of interventions in other settings and contexts is limited by the lack of knowledge of contextual information (e.g., barriers to providing and using the intervention) and resources needed to implement and deliver these interventions [20]. Another process evaluation study of the NUTH e-referral service indicated that identifying contextual factors related to the implementation process and fidelity of the service would facilitate short- and long-term outcome evaluation of the service [21]. In addition, as barriers to providing any intervention might vary depending on the population, setting and context, replication of any service and generalising its outcomes are limited by the context and setting where the service has been implemented [22, 23].

The North-East or NUTH e-referral service, where this study was carried out, is one of the UK’s earliest and longest-sustaining providers of the ToC service. Thus, valuable lessons and factors can be identified from evaluating this service which may differ from any previous evaluation of a similar ToC service. Moreover, the service was launched in the North-East in 2014, but since then, the pharmacy staff has had a low referral rate and uptake of the service [14, 24]. Therefore, the primary aim of this study was to investigate the barriers to the e-referral process in hospital and community pharmacy settings in the North-East of England, and barriers to providing subsequent community pharmacy interventions from the perspectives of the service leaders (SLs), hospital pharmacy staff (HPS) and community pharmacists (CPs). The study also considered a secondary analysis to overcome these barriers by identifying participants’ behaviours that can be modified to improve the provision and use of the e-referral service.

## 2. Methods

### 2.1. Study design

Barriers to providing the e-referral service and delivering community pharmacy services were explored qualitatively via semi-structured face-to-face or telephone interviews with the SLs and service providers, hospital pharmacy staff (HPS), and community pharmacists (CPs). Semi-structured interviews were selected as the appropriate method of investigation to allow flexible exploration of the phenomenon. In addition, one-to-one interviews helped to provide in-depth information of personal barriers for providing or using the e-referral service. The Consolidated criteria for Reporting Qualitative Research checklist (COREQ) [25] has been used to aid transparency in data collection, management, and reporting (S1 Table). All items are detailed within this study except that repeat interviews were not conducted, and transcripts were not returned to participants for feedback or correction.

### 2.2. Participants and recruitment

#### Study settings

Hospital pharmacy staff were recruited from one National Health Service (NHS) Trust (which includes two tertiary hospitals) in the North-East of England. For CPs, the recruitment invitation was sent to all community pharmacies in the North-East (n = 498). However, only nine pharmacists from nine pharmacies have participated in this study. Overall, the interviews with all the key informants took place from 20/07/2018 to 19/08/2019.

#### Sampling procedures

The sampling and recruitment strategies for the interviews with SLs and providers are summarised in Table 1.

**Table 1:** The sampling and recruitment strategies for the interviews with service leaders and providers.

Stakeholder	Sampling strategy	Recruitment strategy
Service leaders	Purposive sampling from the NUTH ToC project team membership.	Participants were known to the research team and invited via email to take part in the study through provision of a participant information sheet and a consent form to participate.
Hospital pharmacy staff	Convenience sampling from the two tertiary hospitals providing the e-referral service in the North-East.	Participants invited by email including a participant information sheet and consent form via a gatekeeper. Invited staff were those who had previously provided the e-referral service to discharged patients.
Community pharmacists	Purposive sampling from all community pharmacies providing the e-referral service in the North-East (n = 498).	Participants were recruited via: • A centralised messaging service. • Face-to-face direct contact in two local events. • Direct phone call & community pharmacy visits. All participants received the participant information sheet and consent form via email.

**Abbreviations:** NUTH ToC, Newcastle-Upon-Tyne Hospitals Transfer of Care.

### 2.3. Data collection

#### Nature and location of the interviews

Semi-structured in-depth interviews were used with all the key informants. Only one interview per person was requested, lasting for a maximum of one hour. During the interviews, the key informants were typically asked several open-ended and probing questions, rather than closed questions, which enabled them to describe their perceptions in their own words. The interviews were held during working hours, in a private room at the participant’s workplace. All interviews were conducted face-to-face, except one telephone interview was conducted with one CP.

#### Development of the interview guide

The topic guides for the SLs, HPS and CPs (published previously [21]) were all informed by the key informant guide by O’Haire et al. [26], the CFIR [27], and a preliminary logic model developed by the research team [21]. The key topics discussed with the SLs were the NUTH e-referral implementation process, and their perception about the use of the service and its quality. While the topic guide with the HPS covered the implementation process, barriers for providing the service, and characteristics of the eToC service (e.g., patient selection criteria, reason for referral and material used in the referral process). For CPs interviews, five key topics were discussed, these are (1) the implementation/operation of the service, (2) the presence of any barriers to using the service and/or to delivering community pharmacy interventions, (3) the characteristics of the community pharmacy interventions, (4) the effectiveness and quality of the service, and (5) service evaluation and potential improvement.

#### Pilot study

The study involved an initial exploratory and piloting phase for all interview guides. In the initial exploratory phase, there were iterative meetings with the research team to discuss the interview guide and amended it accordingly. The interview guide was also sent to a research collaborator who had experience in the e-referral service to identify the validity and applicability of the interview guide. An interview reflection log was used after each interview, to highlight questions that were not clear for the participants or needed additional prompts. The obtained feedback was then discussed with the research team to adapt the interview questions, as appropriate.

#### Transcription

The interviews were audio recorded with informed consent and transcribed verbatim. Transcription was conducted by SMK with some field notes added to the interview reflection log. A standardised anonymisation process was used where each study participant was assigned a unique combination of letters and numbers signifying their participation number and workplace/role, e.g., 02C referred to the second participant in the community pharmacists’ group, 01SL referred to the first participant among the service leaders. Transcripts were quality checked by SMK, which involved listening back to the recording of the interview while checking the transcript for errors or omissions. A second researcher also checked the quality of the transcript. Transcripts were not returned to the participants for comment and/or correction.

### 2.4. Data analysis

In this study, the thematic framework analysis was employed to analyse the data [28]. This framework provided both exploratory and explanatory conclusions required to develop an understanding of the e-referral service and identify barriers to the provision of the e-referral service and delivery of community pharmacy services. The six stages of framework analysis were followed: data familiarisation, coding process, developing an initial thematic framework, applying the thematic framework, reviewing data extracts and developing a framework matrix, and data interpretation [28, 29]. A combination of inductive and deductive coding was used to identify the main themes from the interviews [28]. The analysis was performed using Nvivo12 [QSR International, Melbourne].

The interviews transcription, data familiarisation and identification of main themes and sub-themes were conducted by one researcher (SMK), while another researcher (HN) reviewed the identified themes/sub-themes to validate the findings and resolve any discrepancies. Validity of findings was also enhanced using ‘constant comparative method’ which involved systematic collection, coding and analysis of data [30]. Such a method is important to build the structure and process of the framework analysis method, where data from all three operations are compared simultaneously across study participants as well as within individual participants [28]. The constant comparative process stimulates a researcher’s thoughts leading to both exploratory (descriptive) and explanatory categories/themes [23]. Authors believed that the properties of categories, generated from the constant comparison, would increase the categories’ generality and explanatory power [30]. Moreover, different techniques were used in this study to establish the credibility. These were adoption of appropriate research methods, searching for negative or deviant cases, frequent debriefing sessions, and using tactics to help ensure honesty in informants when contributing data [26]. Participant recruitment continued until saturation was achieved. Details on data saturation and how it was achieved were published elsewhere [21]. In general, a ‘Hybrid’ form of saturation was considered in this study which include data saturation, theoretical saturation, a priori thematic saturation, and inductive thematic saturation. The ‘Hybrid’ form of saturation was previously used by some researchers, as they preferred to combine sampling, data collection, and data analysis rather than treating them as separate stages in a linear process [31, 32].

Ethical approval for interviewing SLs and providers was sought individually and was successfully awarded Institutional ethical approval from the Newcastle University, Faculty of Medical Sciences Ethical Committee (REF 1497/4636), and Research and Design approval from the hospital. The ethical review considered the work not falling in the remit of requiring NHS Research Authority approval.

#### Secondary analysis

A secondary analysis was conducted to identify and address certain behaviours that can be modified to produce the required change to drive better implementation and delivery of the e-referral service. The ‘Behaviour Change Wheel’ (BCW) by Michie *et al*. [15] is the primary model used in this study to identify certain behaviours that can be modified and understand how they impact service provision. The BCW consists of three different layers; the core is related to the COM-B model, which consists of the following three components: ‘Capability’, ‘Opportunity’ and ‘Motivation’ that are all necessary for the given ‘Behaviour’ to occur. The ‘Opportunity’ component is the context of the behavioural change. The other two layers of the BCW are related to nine intervention functions and seven policy categories that were identified from the systematic review performed by Michie *et al*. [33]. Identifying the appropriate intervention function and policy category for certain behaviours would enable policymakers, researchers and service implementers to identify an evidence-based improvement and produce the desired behaviour change [15].

## 3. Results

A total of three SLs, ten HPS (from two hospitals) and nine CPs (from nine different pharmacies) were interviewed; their demographic details are presented in Table 2.

**Table 2:** HPS and CPs’ demographics.

Variables		Hospital pharmacy staff (n = 10)	Community pharmacists (n = 9)
**Age group**	• Mean (SD)	34 (±11)	39 (±10)
	• 18–24 years	2	0
	• 25–34 years	4	3
	• 35–44 years	2	4
	• 45–54 years	1	1
	• 55–65 years	1	1
	• > 65 years	0	0
**Employment status**	• Full-time employed	9	9
	• Part-time employed	1	0
	• Locum pharmacist	0	0
**Occupation**	• Pharmacists	7	9
	• Pharmacy technicians	3	0
**Work experience**	• ≤ 1 year	2	0
	• 2–5 years	2	3
	• 6–9 years	1	1
	• ≥ 10 years	5	5
**Education level**	• Diploma	1	0
	• Bachelor’s degree	3	7
	• Master’s degree	1	0
	• Doctor of Pharmacy	0	1
	• Postgraduate diploma	5	1
**Type of community pharmacy**	• Supermarket pharmacy		0
	• Independent/owned pharmacy (≤5 outlets)		2
	• Small regional pharmacy (≤20 outlets)		2
	• National large chain pharmacy (>20 outlets but <200)		0
	• Multiple pharmacies (≥200 outlets)		5

**N.B:** Service leaders’ demographics were not collected as they would be identifiable to other healthcare professionals working in the field.

On exploring CP’s experiences of providing the e-referral service, it was found that some had used it when it was first introduced in 2014 (n = 6), while others had used it after it had been running for a couple of years (n = 3). Five CPs mentioned that they had worked at different pharmacies and in different locations, either in the same company or in a different one. Their different working places and experiences were considered by the research team, when appropriate, to identify any additional barriers to providing the service.

**Fig 1:**
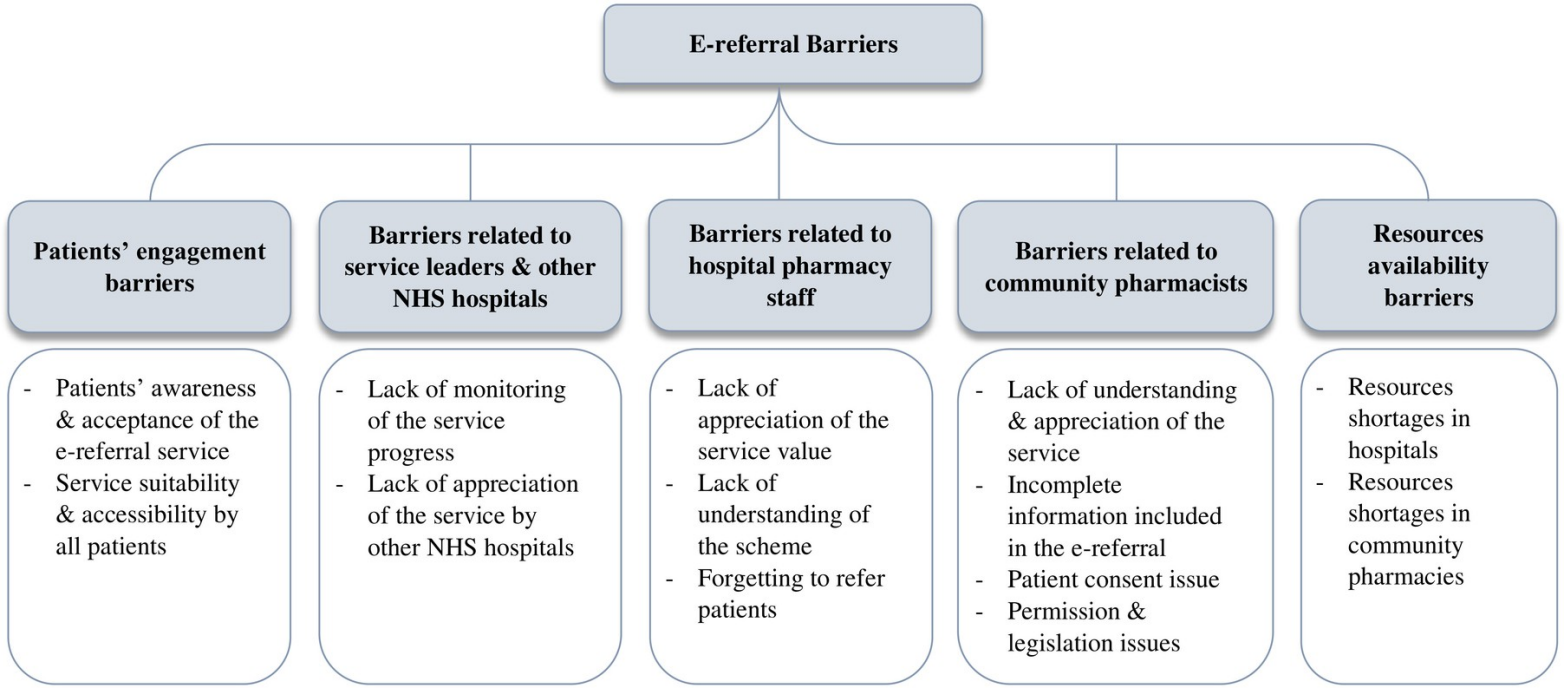
An overview of the barriers to the provision of the e-referral service.

The analysis identified various barriers to the provision of the e-referral service from hospital to community pharmacies in the North-East of England. Some were related to (1) patient engagement (before and after sending the e-referral to the CP), (2) the SLs and other Trusts (i.e., an organisation unit within the National Health Service of England serves a geographical area and is composed of different hospitals), (3) the HPS, or (4) the CPs. Fig 1 provides an overview of these barriers.

### 3.1. Patient engagement barriers

Two main barriers raised by the participants related to patient engagement with the e-referral service, particularly patients’ awareness/acceptance of the e-referral service, and the service suitability and accessibility by all patients. The participants considered that patients did not have enough knowledge and understanding of the value of the e-referral and the benefits of the CP post-discharge services and therefore were not inclined to engage with it. They reported that patients’ awareness of such services depended on their conversation with the service providers and what had been said to the patient whilst in hospital. However, it was believed that most of the e-referrals were for Medibox/compliance aid (information-sharing purpose only) where they did not need to communicate with the patients.

*“Most of our referrals are for patients with Medibox, and because we do not ask their permission, I think a lot of the time they are not even aware that we are sending that information”, 05HPS*

*“The patients did not see the benefits of it all. They just thought it was just something that was going to be a hassle for them when they go home. Especially the less mobile patients, (…) and they always seem to be quite surprised, ohh what is this, and they either vary, no I am not interested in that, or ohh that sounds good”, 03HPS*

The analysis identified different factors contributed to patient poor acceptability of the e-referral service; these were: (1) confidentiality and data sharing, (2) understanding of CP post-discharge services, i.e., participants reported that patient’s perception of the community pharmacist’s role was to supply/sell medicines rather than delivering post-discharge care, (3) response and attitude at the time of discharge, i.e., patients’ negative response to the e-referral service especially those who had an extended hospital stay and did not want any additional medical involvement, and (4) other demographic factors, e.g., the elderly and male patients did appreciate the e-referral service.

*“Patients do not understand who has what information and who has access to what information and this sometimes is a stumbling-block because people do not want others to have certain information and they are now concerned about what information is being shared, and this can sometimes take a little bit more explaining”, 08HPS*

All participants agreed that the e-referral service was not suitable for all hospital discharged patients, such as those who were less mobile, elderly, housebound patients, and those in care homes or with special requirements, e.g., patients with hearing loss

*“At the very start of the process, that was frustration with some patients. You would feel that they would do amazingly well with a little bit of support with their medicines. They just needed their questions answered and to understand and know about the link (e-referral service). But this link was failing because they were housebound, so the service was not there to continue. (..) Community pharmacies are limited in terms of what they can do on the phone as well”, 08HPS*

Hospital pharmacy staff also highlighted that patients had a fast turnover between the hospital departments, e.g., surgical patients, or had a short hospital stay from the time of admission to the discharge point. Therefore, some patients who would be suitable for the service might not be identified by the HPS and offered the e-referral.

*“The turnover of the patients as I have noticed over the last five years is considerably more. Patients were coming in the morning and going home in the afternoon. Patients coming into the assessment ward to transfer to another ward, you do not know where they have gone”, 03HPS*

Similarly, CPs found that it was difficult sometimes to do a post-discharge medicines use review (MUR) (i.e., a structured face-to-face consultation between the pharmacist and patient to discuss and review their medicines) or to provide any other post-discharge care because of the patients’ medical conditions, e.g., some patients might not have the capacity to understand changes in their medicines.

*“Patients could have been in for an operation or had a severe heart attack. They are just not at that stage to come down and discuss their medicines. (..) Nine times out of a ten, it is quite difficult because the patients tend to be housebound”, 02C*

### 3.2. Barriers related to the service leaders and other NHS hospitals

The interviewed SLs reported that the lack of monitoring of the service progress by the SLs, implementers, the Local Pharmaceutical Committee (LPC) members, the pharmacies’ line managers and community pharmacy leaders resulted in a low referral rate.

*“The referrals were quite good at the start but then they kind of dropped off because no one was managing the trade. (..) If no one is monitoring what is going on why bother doing it when there is so many other things to do? (..) The LPC is very much involved in keeping the community pharmacies on their radar, checking out to support them. Our area LPC did that to start with, but I do not think they had the resources at the time to continue”, 03SL*

The interviews with the SLs also revealed that not all NHS hospitals in the area were actively engaged with the service. They either did not appreciate the e-referral service or believed it was only a service provided by the NUTH hospitals rather than a service that could be used in any NHS hospital. Another reason was the negative impression, which contributed to other NHS hospitals lack of motivation to provide the service.

*“The rumours started to other hospitals that it would take ages to send a referral by the time you’ve logged on. Then, community pharmacists are not actually doing anything with them. That sort of general noise probably caused some of the pharmacists at other hospitals to do not engage with the service”, 02SL*

### 3.3. Barriers related to the hospital pharmacy staff

Hospital Pharmacy staff reported some personal barriers related to their provision of the e-referral service, these are HPS appreciation of the service value, understanding of the scheme, and remembering to refer patients. For example, HPS were demoralised to provide the service in the absence of any feedback about its outcome and effectiveness.

*“I do not understand the point in referring because community pharmacists cannot actually do anything”, 06HPS*

*“I do not know what X, Y or Z is at the community pharmacy. What one community pharmacy can offer might not be what another pharmacy can offer. There could be a cohort of patients that community would love to get referred but we are not referring them because we do not know the community would love to get them referred”, 07HPS*

On some occasions, remembering to refer patients was also a barrier to provide the e-referral service.

*“We have had incidences where the e-referral has not been sent (..) the patient has come back into the hospital a couple of weeks later because the medication had not been stopped. Someone meant to have done it but it just slipped their mind”, 05HPS*

### 3.4. Barriers related to the community pharmacists

The interviews with the CPs revealed some barriers to improving patient care and clinical outcomes after sending the e-referral request; these were: (1) CPs’ lack of understanding and appreciation of the service, (2) incomplete information included in the e-referral, (3) patient consent issue, and (4) permission and legislation issues in community pharmacies.

According to the participants, when the service was initiated, there was a gradual uptake by CPs in their acceptance and understanding of the service. However, some still preferred to use the old method of communication, namely, faxing the discharge letter. In addition, the SLs agreed that less than half of the received e-referrals were completed by the CPs, where they counselled the patients and reviewed their medication.

*“Another comment that kept been picked up by the Trust is that community pharmacies are only doing a third. Why am I bothering to send them if they’re only completing a third of the referrals?. (..) Community pharmacists maybe are not understanding the impact that they can have and the benefit that they can actually give to patients”, 02SL*

One of the SLs also indicated that multiple community pharmacies had the highest engagement levels and pick-up rate of e-referrals, while other pharmacies did not engage with the service despite the LPC’s efforts to advertise it. Both SLs and CPs highlighted that some CPs forgot to follow-up the e-referrals because it was not always a top priority (CPs had competing priorities).

*“Sometimes it is like, ‘Oh gosh, referrals coming through in my email saying I have not looked at them’ and I need to, but you forget. I’ll be honest with you, it is not the first thing on your mind all the time”, 02C*

In addition, most interviewed CPs believed that patients who had a new medicine during their hospital stay would receive the required care, e.g., new medicines service (NMS), at the hospital or from the general practitioner (GP) or practice pharmacist who works in the GP centre. Therefore, there was no need for further CP support.

*“If patients started their medicine in hospital we are not supposed to do NMS because they have already started it”, 07C*

CPs indicated that, due to the lack of information included in the e-referral request, some were unable to provide optimum care to the referred patients. For example, sending incomplete discharge medication lists prevented CPs from identifying any drug-related problem or changes in medication during the hospital stay.

*“That (sending incomplete medication lists) will be half of the picture. Community pharmacists will not be able to identify any drug interactions or anything that has been stopped or started. There won’t be any point in sending community pharmacists half of the information”, 05HPS*

Both the SLs and HPS indicated that the referring staff were only obtaining patients’ agreement on the e-referral; they did not obtain patients’ consent to receive any community pharmacy post-discharge care. Therefore, interviewed CPs highlighted the need for the patient to give written consent to enable them to provide the required post-discharge services.

*“If the patients have consented to having their transfer of care sent to their pharmacy and are simultaneously consenting to get a phone MUR, within that process we have then got consent to do that. No problem. It is just the consent”, 01C*

However, some interviewed CPs did not have any problem with getting patient consent to provide community pharmacy post-discharge services because they were sending the driver to the patient’s home to get their signed consent.

The interviews with CPs also revealed that they were limited in terms of the services they provided over the phone especially for housebound patients. For instance, to provide domiciliary services or other services over the phone, they needed to follow the NHS legislation and get the required permissions, i.e., CPs were required to apply to NHS England each time they wish to conduct an MUR in the patient’s home. However, the NHS permissions and legislation were described as ‘red tape’ to provide the required post-discharge care. Therefore, CPs suggested that there is now a need to change the legislation or the e-referral service specifications to enable them to provide more services over the phone or to do domiciliary visits.

### 3.5. Resources availability barriers

Participants reported a shortage in the resources they had in hospital and community pharmacy settings, which limited the provision of the e-referral service to the needed patients. Generally, HPS believed that they needed recap meetings and refresher training to provide the service. This was mainly recommended for new/junior pharmacy staff, when there is staff turnover, and for people who had been away from practice.

*“That is a bit of a gap in our practice (…), maybe we do not use it as well as we should because we have not had a refresher to say these are the people you should be targeting”, 07HPS*

Staff shortage and turnover were also barriers for HPS, especially when hospital wards had no pharmacy coverage or there was an overload on the pharmacists in particular wards that would affect the number of their e-referrals.

*“The shortage of the staff is probably the main barrier to effective use of the service,(..) till we are better staffed, I do not think the quantity of referrals will improve. (..) Because it is not a priority, it will depend on how many wards I was covering on that day. So some patients might get a referral, another patient might not”, 05HPS*

Community pharmacists also reported having time-pressure/workload in the pharmacies, especially in central or busy areas, which hindered them from delivering any post-discharge care to the referred patients.

*“To go on the system all the time, to look at the communication could be hard sometimes because we are busy”, 04C*

Community pharmacy staff availability and turnover was also another important barrier, especially when providing home MUR was required.

*“There are barriers that currently you cannot overcome. If I had a second pharmacist here and one of us could go to see the patient while I run the pharmacy, then that would be fine”, 08C*

### 3.6. Findings of the secondary analysis

Three COM-B model sub-components were related to interview data; these are physical opportunity, reflective motivation, and psychological capability. The physical opportunity included barriers related to environmental context (service suitability/accessibility) and the availability of the resources. The reflective motivation included barriers related to the participants’ beliefs of the service value, its outcomes and quality. The psychological capability included barriers related to service providers’ understanding (knowledge) of the e-referral service, and behavioural regulation (i.e., the availability of mechanisms to monitor/evaluate the e-referral service).

## 4. Discussion

This study presents findings from the interviews with the SLs and providers related to the context and mechanism of impact of the e-referral service to improve patient care. Various barriers were identified in the study; some were related to the referred patients (e.g., their lack of awareness and acceptance of the e-referral service), the healthcare professionals (e.g., HPS and CPs’ lack of appreciation of the service) or the availability of resources (e.g., the lack of refresher training on service provision). Identifying these barriers can help SLs, service implementers and policymakers identify areas for service optimisation which could result in increased service adoption, effectiveness and sustainability.

### Physical opportunity

#### 1. Environmental context

This study found that the e-referral was not suitable for less mobile patients (e.g., patients who had undergone recent surgery), housebound patients or those with special requirements. The uncertainty of discharged patients in their ability to attend the community pharmacy visits for post-discharge care was reported as a barrier by various studies [34–36]. One of the implemented solutions by the Royal Pharmaceutical Society (RPS) for the accessibility problem was to provide domiciliary or phone MURs (‘Enablement’ intervention function as per the BCW model) [16]. However, according to CPs in this study, these solutions were not practical due to the difficulty in obtaining the patient’s consent. This was similar to the findings by Hodson *et al*., who found that obtaining patient’s consent to deliver the Discharge Medicine Review (DMR) service was considered as a barrier by 38% of responding CPs in an electronic survey [17]. In the same evaluation, CPs suggested having a blanket consent form for all of their services with consideration given to the duration of the validity for that consent [17]. According to the BCW model, the policy category that should be considered in this case is changing the service guidelines/regulation [15].

#### 2. Availability of the resources

Hospital pharmacy staff and CPs in this study indicated that their appreciation/provision of the service was compromised when they had insufficient resources. Previous studies have similar findings; time-pressure/workload and staff shortages in hospitals [17, 37] and community pharmacies [17, 38] were major resource-related barriers to the ToC and to providing post-discharge services. The lack of trained referring HPS who had sufficient knowledge of the discharge process, community services and the referral criteria was another important barrier to the continuity of patient care in this study and a previous one [37]. In contrast, CPs in this study did not have any concerns related to the need for additional training, unlike findings from the literature [18, 19]. For example, the evaluation of the NHS 111 referral to the community pharmacy showed that 47% (n = 104) of participating CPs reported the need for further training on the use of the e-referral platform [18]. The difference in the CPs’ perspective between this evaluation and the current one could be attributed to the time-frame between both evaluations, in which the use of the e-referral platform has become more embedded in the CPs’ routine work. According to the literature and BCW, ‘environmental restructuring’ (i.e., changing the physical or social context) is the most appropriate intervention to overcome issues related to resources [15, 37]. This should be considered with changing the guidelines and environmental/social planning (i.e., overcome the resources limitations) as appropriate BCW policy categories. For example, to overcome the workload and staff shortages during hospital discharge, Bowles *et al*., recommended: (1) reviewing hospital professional roles, policies and procedures pertaining to the discharge process, (2) using a simple and standardised process of sharing the discharge information, and (3) investing in technology, e.g., the use of electronic and automated methods of communication [37].

### Reflective motivation

Similar to this study, the DMR evaluation discovered that 52% of the participating HPS had competing priorities, making it difficult for the service to be their primary concern, particularly when they had staff shortages [17]. In our study, HPS poor appreciation of the value of the e-referral service was principally related to their lack of knowledge of any clear evidence/feedback concerning the service impact and outcomes, which was also reported in other similar ToC evaluations [17, 19]. At the time of conducting this study, there were some published papers related to the outcomes of the ToC services in the UK [24, 34, 39–41]. However, only the evaluation of the NUTH e-referral service [24] was recognised by a few HPS participants in this study. As recommended by the RPS, to ensure successful implementation and elevate service providers’ motivation, SLs and/or service implementers need to provide educational and training programmes which describe the need for the e-referral service and benefits to the patients with the use of evidence to justify this need and demonstrate good practice [16]. This is in line with the BCW recommendations, in which ‘Education’ was the appropriate intervention function to overcome barriers related to the reflective motivation [15]. Authors believed that such educational/training programmes could be enriched by providing meaningful and memorable stories (the ‘Modelling’ intervention function in the BCW) which demonstrate the impact of the referral process on patient care [15, 42, 43].

Having a two-way communication system between hospital and community pharmacies was also perceived as being important in sustaining the e-referral service and in maintaining staff interest and motivation [19]. The RPS e-referral toolkit indicated that CPs can feedback to the hospital the outcome of their intervention [16]. However, sending such feedback to the HPS was not mandatory for the CPs and not mentioned in the e-referral service standards [16] (The Newcastle upon Tyne Hospitals NHS Foundation Trust [Unpublished]). According to the BCW framework, the two-way communication system is appropriate to change the current practice as it involves two intervention functions, namely, ‘Persuasion’ (e.g., discussing positive/negative CPs feedback with HPS to simulate action) and ‘Modelling’ (e.g., CPs providing HPS with reports/quantitative data that prove the service effectiveness to the patients). Both intervention functions should be considered with the use of ‘communication’ as the appropriate BCW policy category [15]. According to Ferguson *et al*., the two-way communication system has recently been applied with the use of an e-mail auto-generated by the R2P system when a referral request has been completed by a CP. The e-mail provides a summary of the e-referral outcome and other essential information such as the presence of prescribing errors [19].

### Psychological capability

#### 1. Service providers’ knowledge of the e-referral service

Comparable to this study, many studies found that the use of discharge services depended upon HPS knowledge of the service and their engagement to identify patients need for post-discharge care [14, 17, 44]. For example, Hodson *et al*. reported inadequate engagement of HPS promoting and offering the DMR service to inpatients due to their lack of awareness and understanding of the service (rated as a major barrier by 42% of respondent HPS) [17]. Moreover, this study found that CPs had a lack of understanding of the e-referral service and post-discharge clinical services which impacted on their level of engagement with the e-referral service. For example, most CPs in this study expected that discharged patients with a new medicine had received the required counselling by other healthcare professionals. However, the NMS is a nationally commissioned community pharmacy service that has been established as an advanced role for CPs in the management of medicines use [13]. Following the BCW, the appropriate way to improve the service providers’ psychological capability and embed the e-referral service into their existing practice would be to provide them with educational and training programmes (via ‘communication/marketing’ BCW policy category) [15]. For example, some authors recommended including content on discharge planning and referral in the hospital orientation and health professionals’ curricula [37]. Others recommended providing local and collaborative learning meetings between primary and secondary care providers involved in the discharge process, to understand each other’s competencies and increase awareness of post-discharge services [42].

#### 2. Behavioural regulation (service monitoring/evaluation)

Similar to the evaluation of the R2P service [19], this study ascertained that there was a lack of service process evaluation and of evaluating the outcomes of the e-referrals which was regarded as a gap in the current practice. Previous studies stressed the need to have strong leadership in both hospital and community pharmacy settings to ensure successful implementation and promote staff motivation and engagement with the scheme [14, 19]. This would be achieved by supporting the staff in educational/training events to build their professional confidence (e.g., improve their counselling skills) and overcome barriers to the new practice [14, 19]. Such strategies are in line with the BCW recommendations in which ‘Education’, ‘Training’ and ‘Enablement’ were the intervention functions that were likely to be effective in creating the change. The appropriate policy categories to support these intervention functions are communication/marketing, guidelines and environmental/social planning [15].

Contexts and implications for practice identified from this study can be iteratively tested across further sites where the e-referral service has been implemented. In England, a recent rapid review found ten studies related to ToC interventions that could be considered as other sites for further evaluation and development of a programme theory [6]. Findings from this process will add richness and granularity to the theory but also highlight aspects of the context, intervention and mechanism that are adaptable or inflexible. This will mean the subsequent evidence-based recommendations that can be made to intervention designers, commissioners and implementers are more specific in relation to achieving successful implementation and positive patient outcomes. This bears significant relevance in England at this time, where the new national DMS has been commissioned [7]. The design, implementation and delivery of this new service can capitalise on the learning from the findings of this study to improve the potential for success and sustainability, especially since the DMS was designed and rolled out nationally without an in-depth evaluation. Moreover, findings of this study are likely to help inform evidence-based recommendations to avoid and address potential challenges in the system when implementing and evaluating national post-discharge services based on the same principle, such as the DMS.

### Strengths and limitations of the study

This study had two strengths; firstly, it provided a comprehensive evaluation from the perspectives of all key informants (SLs, HPS and CPs) who were involved in the service provision and delivery, which in turn is useful for other healthcare professionals who are considering adopting similar post-discharge services [19]. In addition, we previously conducted a similar service evaluation with patients and the public to explore their perspectives and barriers to using the e-referral service [45]. Secondly, many researchers believed that electronic innovations, such as the e-referral service, should undergo a rigorous evaluation throughout all stages of the technology’s life-cycle prior to its large-scale implementation, because this would help to identify which factors were likely to maximise successful implementation [9, 19, 46]. Considering this, the current study had the advantage of being undertaken after the scheme was introduced and used for around six years where the entire stages of the implementation process were evaluated to identify any potential barriers to the provision of the service.

Due to the nature of qualitative research, findings may be of limited generalisability and may differ in other contexts and settings. However, a ‘thick description’ of the fieldwork context was considered, published previously [21], to ensure the credibility and transferability of the findings. This study was carried out in a single NHS Trust in the North-East which was the first Trust to introduce the e-referral service, and while valuable lessons can be learnt from this single case study, it is important to bear in mind that the implementation of this service may differ in other Trusts. In addition, even though different recruitment methods were used to recruit CPs, it may be likely that the interviewed participants were more engaged and positive towards the service than those who did not volunteer to be interviewed. It may be the case that pharmacists who did not volunteer may be less aware and engaged with the service, and therefore may have provided a differing perspective.

## 5. Conclusion

This study provided an in-depth understanding of barriers related to the provision of the e-referral service which is essential to drive improvement and facilitate wider diffusion and adoption of the service at other sites. Moreover, the use of implementation science and BCW model as a lens to assess this service enables the identification of certain behaviours that can be modified to produce the required change to drive better implementation and delivery. The key recommendations to optimise the current service and inform future service implementers include (1) ensuring all key informants are fully aware of the aim and value of the service; (2) sharing leadership, responsibility and accountability of the service between hospital and community pharmacy; (3) integrating a two-way communication system to transfer information/feedback; (4) providing regular opportunity for training, learning from the service and interaction between the two sectors; and (5) sharing understanding of the existing environmental barriers, so that shared strategies to address/overcome them are sought.

## Supporting information

S1 Table The Consolidated criteria for Reporting Qualitative Research checklist (COREQ). (PDF)

S1 File Inclusivity in global research. (DOCX)
