# Supplementary material for: Qualitative investigation of barriers to providing an electronic hospital to community pharmacy referral service for discharged patients
Source: PLoS One. 2023 Mar 31;18(3):e0283836. doi: 10.1371/journal.pone.0283836 (PMC10065289; doi:10.1371/journal.pone.0283836)
Supplement: S1 Table — (PDF) [file pone.0283836.s001.pdf]

Supplementary file 1: Consolidated criteria for reporting qualitative studies (COREQ).

No.	Item	Description	Section #
**Domain 1: Research team and reflexivity**			
**Personal characteristics**			
1.	Interviewer/ facilitator	Which author/s conducted the interview or focus group?	SMK
2.	Credentials	What were the researcher's credentials?	MSc
3.	Occupation	What was their occupation at the time of the study?	PhD student
4.	Gender	Was the researcher male or female?	Female
5.	Experience and training	What experience or training did the researcher have?	SMK has attended different training sessions/courses to support the effective delivery of the interviews. SMK was also seeking support from the supervisory team, who have extensive experience of interviewing participants
**Relationship with participants**			
6.	Relationship established	Was a relationship established prior to study commencement?	SMK had no prior relationships with any of the participants
7.	Participant knowledge of the interviewer	What did the participants know about the researcher?	None of the participants knew the interviewer prior to the interview
8.	Interviewer characteristics	What characteristics were reported about the interviewer/facilitator?	No characteristics reported
**Domain 2: Study design**			
**Theoretical framework**			
9.	Methodological orientation and theory	What methodological orientation was stated to underpin the study?	Thematic framework analysis
**Participant selection**			
10.	Sampling	How were participants selected?	Purposive and convenient sampling technique
11.	Method of approach	How were participants approached?	E-mail (all participants)<br>Face-to-face/telephone (CPs)
12.	Sample size	How many participants were in the study?	3 SLs, 10 HPS and 9 CPs
13.	Non-participation	How many people refused to participate or dropped out? What were the reasons for this?	No participants dropped out
**Setting**			
14.	Setting of data collection	Where was the data collected?	Meeting room in a university; tertiary hospitals; community pharmacies; and over the telephone

No.	Item	Description	Section #
15.	Presence of non-participants	Was anyone else present besides the participants and researchers?	No
16.	Description of sample	What are the important characteristics of the sample?	See Table 2
**Data collection**			
17.	Interview guide	Were questions, prompts, guides provided by the authors? Was it pilot tested?	Yes – See Development of the interview guide and Pilot study sections
18.	Repeat interviews	Were repeat interviews carried out? If yes, how many?	No
19.	Audio/visual recording	Did the research use audio or visual recording to collect the data?	Audio recording
20.	Field notes	Were field notes made during and/or after the interview?	Yes – after each interview
21.	Duration	What was the duration of the interviews?	The average length of the interviews was 47 ±14 mins
22.	Data saturation	Was data saturation discussed?	Yes – under data analysis
23.	Transcripts returned	Were transcripts returned to participants for comment and/or correction?	No
**Domain 3: analysis and findings**			
**Data analysis**			
24.	Number of data coders	How many data coders coded the data?	One (SMK)
25.	Description of the coding tree	Did authors provide a description of the coding tree?	Yes – See Figure 1
26.	Derivation of themes	Were themes identified in advance or derived from the data?	A combination of thematic inductive and deductive analysis was employed (published elsewhere (13))
27.	Software	What software, if applicable, was used to manage the data?	Nvivo12 computer software
28.	Participant checking	Did participants provide feedback on the findings?	No
**Reporting**			
29.	Quotations presented	Were participant quotations presented to illustrate the findings? Was each quotation identified?	Yes
30.	Data and findings consistent	Was there consistency between the data presented and the findings?	Yes
31.	Clarity of major themes	Were major themes clearly presented in the findings?	Yes
32.	Clarity of minor themes	Is there a description of diverse cases or discussion of minor themes?	Yes
